# The Genetic Characterization of a Novel Natural Recombinant Pseudorabies Virus in China

**DOI:** 10.3390/v14050978

**Published:** 2022-05-06

**Authors:** Jianbo Huang, Wenjie Tang, Xvetao Wang, Jun Zhao, Kenan Peng, Xiangang Sun, Shuwei Li, Shengyao Kuang, Ling Zhu, Yuancheng Zhou, Zhiwen Xu

**Affiliations:** 1 College of Veterinary Medicine, Sichuan Agricultural University, Chengdu 611130, China; jianbohuang90@outlook.com (J.H.); zhaojunjoy@126.com (J.Z.); kenan-peng@outlook.com (K.P.); sun.xian.gang@163.com (X.S.); abtczl72@126.com (L.Z.); 2 Livestock and Poultry Biological Products Key Laboratory of Sichuan Province, Animtech Bioengineering Co., Ltd., Chengdu 610299, China; wenhan28@126.com (W.T.); lishuwei84511614@126.com (S.L.); ksy_cd@163.com (S.K.); 3 Veterinary Biologicals Engineering and Technology Research Center of Sichuan Province, Animtech Bioengineering Co., Ltd., Chengdu 610066, China; 15983342580@163.com; 4 Key Laboratory of Animal Diseases and Human Health of Sichuan Province, Chengdu 611130, China

**Keywords:** pseudorabies virus, complete genome sequencing, phylogenetic analysis, gene recombination

## Abstract

We sequenced the complete genome of the pseudorabies virus (PRV) FJ epidemic strain, and we studied the characteristics and the differences compared with the classical Chinese strain and that of other countries. Third-generation sequencing and second-generation sequencing technology were used to construct, sequence, and annotate an efficient, accurate PRV library. The complete FJ genome was 143,703 bp, the G+C content was 73.67%, and it encoded a total of 70 genes. The genetic evolution of the complete genome and some key gene sequences of the FJ strain and PRV reference strains were analyzed by the maximum likelihood (ML) method of MEGA 7.0 software. According to the ML tree based on the full-length genome sequences, PRV FJ strain was assigned to the branch of genotype II, and it showed a close evolutionary relationship with PRV epidemic variants isolated in China after 2011. The gB, gC, gD, gH, gL, gM, gN, TK, gI, and PK genes of the FJ strain were assigned to the same branch with other Chinese epidemic mutants; its gG gene was assigned to the same branch with the classic Chinese Fa and Ea strains; and its gE gene was assigned to a relatively independent branch. Potential recombination events were predicted by the RDP4 software, which showed that the predicted recombination sites were between 1694 and 1936 bp, 101,113 and 102,660 bp, and 107,964 and 111,481 bp in the non-coding region. This result broke the previously reported general rule that pseudorabies virus recombination events occur in the gene coding region. The major backbone strain of the recombination event was HLJ8 and the minor backbone strain was Ea. Our results allowed us to track and to grasp the recent molecular epidemiological changes of PRV. They also provide background materials for the development of new PRV vaccines, and they lay a foundation for further study of PRV.

## 1. Introduction

Pseudorabies (PR), also known as Aujeszky’s disease (AD), is an acute infectious disease caused by the pseudorabies virus (PRV) [1]. The disease can infect many livestock species and wild animals [2]. Pigs are the main vector for the virus. One of the main symptoms of diseased pigs is an elevated body temperature. In addition, newborn piglets mainly show neurological symptoms of encephalomyelitis, which can also invade the digestive system. Adult pigs often show recessive infection; pregnant sows can have miscarriages, stillbirths, and mummified fetuses; and boars show reproductive disorders and dyspnoea [3]. The disease is distributed all over the world, and it has been eradicated in the United States, Germany, the United Kingdom, Denmark, and the Netherlands. In other countries, it is one of the major diseases that greatly harms the swine industry [4,5]. The World Organization for Animal Health (OIE) lists pseudorabies as a notifiable infectious disease. In China, pseudorabies is classified as a second-class animal epidemic.

The PRV virus is a member of the Herpesviridae, Alphaherpesvirinae subfamily, varicella virus genus [6]. In terms of genome structure, PRV consists of a unique long region (UL), a unique short region (US), and a terminal repeat sequence (TR) and an internal repeat sequence (IR) at both ends of the US region [5]. At present, there is only one PRV serotype, and its genome is composed of double-stranded DNA, the length of which is approximately 143 kilobase pairs (kbp); the GC bases content can be as high as 74%. It contains at least 70 open reading frames (ORF) of which more than 50 proteins are structural; they can participate in the formation of viral capsid, tegument, and the envelope structure [7,8].

In the 1970s, the PRV Bartha-k61 vaccine strain was imported into China, and pseudorabies was well controlled for a time [9,10]. However, since 2012, outbreaks of porcine pseudorabies have been reported in many areas of China, and they have seriously endangered the development of the swine industry [11,12]. These outbreaks are due to the emergence of mutated PRV in various parts of China. The amino acid sequences of important glycoproteins such as gB, gC, gD, and gE have changed, and the existing Bartha-k61 vaccines can no longer elicit 100% protection against mutated PRV strains. It is imperative to develop new PRV vaccines. So, several years ago, PRV was divided into two distinct clusters with the gC gene used as the criterion, with Chinese strains classified as genotype II and PRVs isolated from Europe and North America classified as genotype I [13]. To track the genetic variation of PRV in China, we recently sequenced the complete genome of the PRV FJ strain isolated and identified from the brain tissue of suckling piglets in a pig farm in Fujian province. The genetic relationship between this strain and PRV strains in China and abroad were revealed by a series of bioinformatic analyses so as to provide data support for the development of a new genetically engineered PRV vaccine.

## 2. Materials and Methods

### 2.1. Isolation of PRV FJ Strain

Recently, pseudorabies broke out at an intensive pig farm in Fujian province, China. Some suckling piglets and weaned piglets had fever, lethargy, neurological symptoms, and they died. Sows gave birth to stillbirths and weak fetuses. The staff immunized pigs with PRV vaccine by the method of nasal drops six months ago. The incidence rate was approximately 23%, and the death rate of piglets was over 14% on the pig farm. We isolated and identified a PRV strain from 35 brain and tonsil samples of suckling piglets brought from the farm, and we named it the PRV FJ strain. After many rounds of virus multiplication and plaque purification in cell culture flasks, fluid virus samples were collected and frozen in a cryogenic refrigerator at −80 °C.

### 2.2. Concentration and Purification of the Virion

The PRV FJ strain was inoculated into a full monolayer of BHK-21 cells. After the cytopathic effect (CPE) reached 80−90%, the cell culture flask was placed in a cryogenic refrigerator at −80 °C. After freezing and thawing three times, the cell culture flask was separately packed into a 50 mL aseptic centrifuge tube, and the supernatant was centrifuged at 4 °C and 3500 rpm for 15 min. After the supernatant was sterilized and filtered by a 0.45 μm filter membrane (Millipore, Billerica, MA, USA), it was transferred to a 15 mL ultrafilter tube with a maximum cut-off of 100 kD (Millipore, Billerica, MA, USA). The tube was centrifuged at 4000× *g* for 30 min according to the manufacturer’s instructions. After centrifugation, the concentrated liquid on the filter membrane was carefully aspirated with a 2–200 μL range pipette (Eppendorf, Hamburg, HAM, Germany).

### 2.3. DNA Extraction

DNA of the PRV FJ strain was extracted using the phenol–chloroform method:

(1) 200 μL of the 10% SDS solution, and 15 μL of the 10 mg/mL RNase A were added in the Eppendorf tubes and incubated at 60 °C for 30 min in the metal bath (Cole-Parmer, Chicago, IL, USA).
(2) 100 μL of the 10 mg/mL Proteinase K was added and incubated in a metal bath at 56 °C for 30 min.
(3) The ddH_2_O was added to make up the concentrated viral solution to 400 μL, then 600 μL of phenol: chloroform: isoamyl alcohol = 25:24:1 DNA extraction reagent was added, the Eppendorf tubes were carefully inverted and mixed, then the Eppendorf tubes were stood for 5 min to make the liquid stratified.
(4) These Eppendorf tubes were centrifuged for 5 min at 12,000 r/min with a microcentrifuge (Thermo Scientific, Waltham, MA, USA), and they took the supernatant to avoid aspirating to the impurities in the middle layer.
(5) Repeat steps (3) and (4).
(6) An equal volume of isopropanol was added, mixed lightly, and precipitated for 1 h in a refrigerator at −20 °C.
(7) These Eppendorf tubes were centrifuged at 12,000 r/min for 5 min with the microcentrifuge to discard the supernatant, 800 μL of anhydrous ice ethanol was added, then 1/10 volume of 3 mol/L NaAc was added, washed with light mixing, and left for 5 min.
(8) These Eppendorf tubes were centrifuged at 12,000 r/min for 5 min with the microcentrifuge at 4 °C to discard the supernatant, the precipitate was placed in a biosafety cabinet, the exhaust air was turned on and blown until there was no smell of alcohol.
(9) The precipitate was carefully dissolved in 100μL TE solution.

Then the extracted DNA samples were stored in a refrigerator at −20 °C for further use.

### 2.4. Sequencing the Complete Genomes

The extracted PRV FJ DNA samples were sent to Wuhan BaiYi biotechnology company for complete genome sequencing. After the samples were qualified, the database was built with the PRV HLJ8 strain (National Center of Biotechnology Information [NCBI] accession number: KT824771.1) as the reference sequence. Third- and second-generation high-throughput sequencing was carried out using a PacBio RS II sequencing system and a MGISEQ-2000 sequencing system, respectively. For the PacBio RS II system, the Sequel Binding Kit 2.1, the Sequel Sequencing Kit 2.1, and the Sequel SMRT Cell 1mv2 (Pacific Biosciences, Menlo Park, CA, USA) were used for sequencing. The data were processed with the SMRT LINK 6.0 software. The read quality value in the original data was filtered. Based on the complete genome sequencing using the PacBio equipment, the obtained sequence was corrected using the MGISEQ-2000 s-generation sequencing platform. Finally, the complete PRV FJ genome sequence was assembled and annotated.

### 2.5. Genome and Related Gene Homology and Phylogenetic Analysis

Fifteen PRV genome sequences uploaded to NCBI (Table 1) were compared with the PRV FJ genome sequence and its major virulence, glycoprotein, and immunogenicity-related coding sequences(CDS): TK, PK, gB, gC, gD, gG, gH, gL, gM, gN, gI, and gE. A genetic evolution tree was drawn and analyzed using the MEGA 7.0 software (https://www.megasoftware.net, accessed on 25 October 2021).

### 2.6. Prediction of Potential Genome Recombination Events

The genome alignments from the 15 PRV reference strains and the FJ strain were analyzed with the Recombination Detection Program 4 (RDP4) software to screen for potential recombination events. Seven algorithms, including RDP, BootScan, GENECONV, Maxchi, SiScan, Chimera, and 3Seq were employed [14].

### 2.7. Sequence Submission

The complete PRV FJ genome sequence was deposited in the GenBank database (http://www.ncbi.nim.nih.gov/genbank, accessed on 18 October 2021) under the accession numbers of MW286330.

## 3. Results

### 3.1. Complete Genome Sequence Analysis

**Figure 1:**
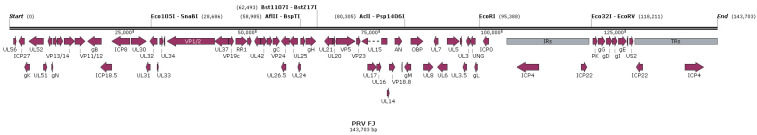
Sequences and distribution of the PRV FJ complete genome.

After comparing the genome sequence assembly using the reference PRV sequences from NCBI databases, the PRV FJ complete genome length was 143,703 bp, the GC bases content was 73.67%, and it encoded a total of 70 genes without insertion and deletion of the rest of the coding sequences. The sequence was divided into four parts: UL, US, IRs, and TRs (Table 2). We annotated the linear map, gene arrangement, and distribution of the complete genome sequence using the Snapgene software (Figure 1), and we noted the annotations of each open reading frame (ORF) (Table 3).

### 3.2. Genomic Genetic Evolution Analysis

Nucleotide homology comparison between the FJ strain and the reference strains using the MEGA 7.0 software showed that the FJ strain had the highest homology with Chinese PRV mutant strains isolated after 2011, with 99.9% and 99.7% homology with classical PRV Fa and Ea strains, respectively, isolated in the 20th century in China. The homology with the other country’s MY-1, Bartha, Becker, Kaplan, and Kolchis strains was 99.0%, 95.7%, 95.7%, 96.0%, and 95.7%, respectively; these values are relatively low (Table 4). It is worth noting that the MY-1 strain is an Asian strain.

**Figure 2:**
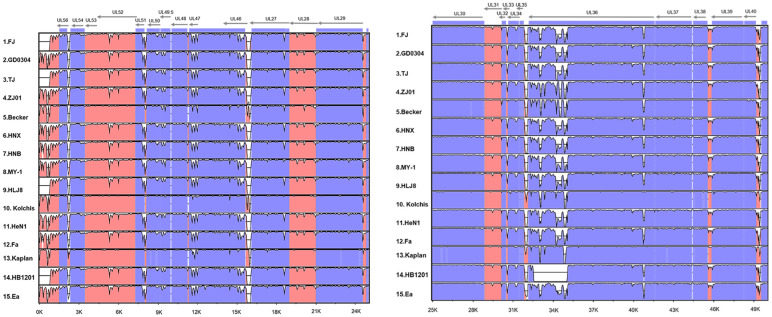

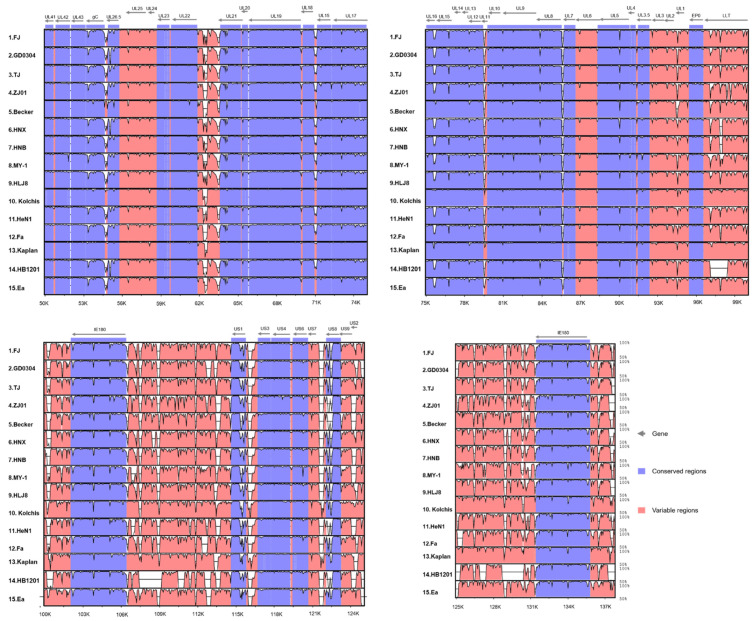
Genome organization and comparison of the PRV Bartha strain with the PRV FJ strain and the remaining 14 reference PRV strains. Comparison of the PRV genome shows conserved (blue) and variable (pink) regions. Open reading frames (ORFs) are represented by gray horizontal arrows across the top of each panel, and genome coordinates in kilobase pairs (kbp) are shown along the bottom.

We analyzed the complete genome sequence homology of the reference strains and the FJ strain with the online program mVista (http://genome.lbl.gov/vista/mvista/submit.shtml, accessed on 3 October 2021). Compared with the Bartha strain, the FJ strain had low homology in UL56, UL51, UL27, UL36, UL41, UL28.5, UL21, and LLT genes. Except for the HB1201 strain, the other Chinese reference strains and the MY-1 strain also showed homology differences in the above regions. The other country’s Becker, Kolchis, and Kaplan reference strains only showed significant homology differences in UL27, UL36, UL21, and US1 gene regions. The homology difference between HB1201 and Bartha was the greatest, and there were large base deletions in the UL56, UL27, UL21, UL36, LLT, US1, US3, and IE180 gene regions. In summary, after homology comparison with the Bartha strain, the regions with lower homology between the FJ strain and the other reference strains were mainly distributed in the non-coding region (Figure 2).

**Figure 3:**
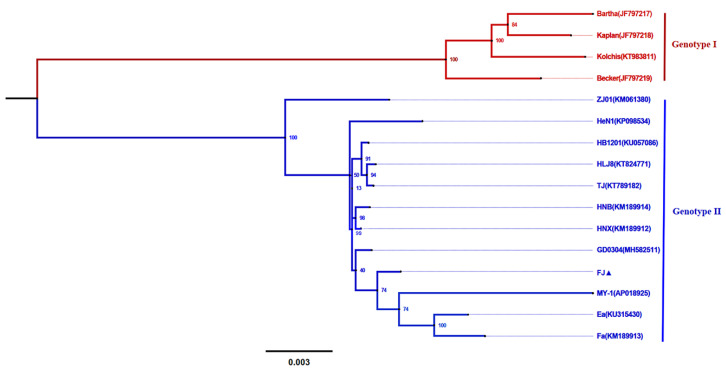
Phylogenetic analysis of the PRV complete genome sequences. The tree was constructed using the MEGA 7.0 software with the maximum likelihood method and 1000 bootstrap replicates. The bar and the number represent the genetic distance scale of these genes at this length is 0.003.

We constructed and analyzed the genetic evolution tree of the complete genome sequence of PRV strains with the maximum likelihood (ML) method. All the strains were classified into two major branches. The other country’s Bartha, Becker, Kolchis, and Kaplan strains were located in the European and the North American genotype (genotype I) branch, while the Chinese strains and the MY-1 strain were located in the Asian genotype (genotype II) branch, which was consistent with the results of other reported genetic evolution analyses. The FJ strain was still located in the genotype II branch, close to the GD0304 strain branch, and it had the lowest genetic relationship with the ZJ01 strain in the genotype II branch (Figure 3).

### 3.3. Phylogenetic Analysis of Related Gene Sequences

**Figure 4:**
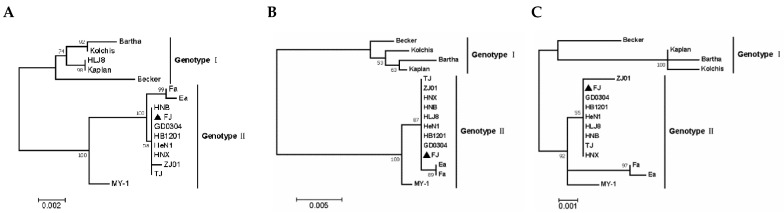

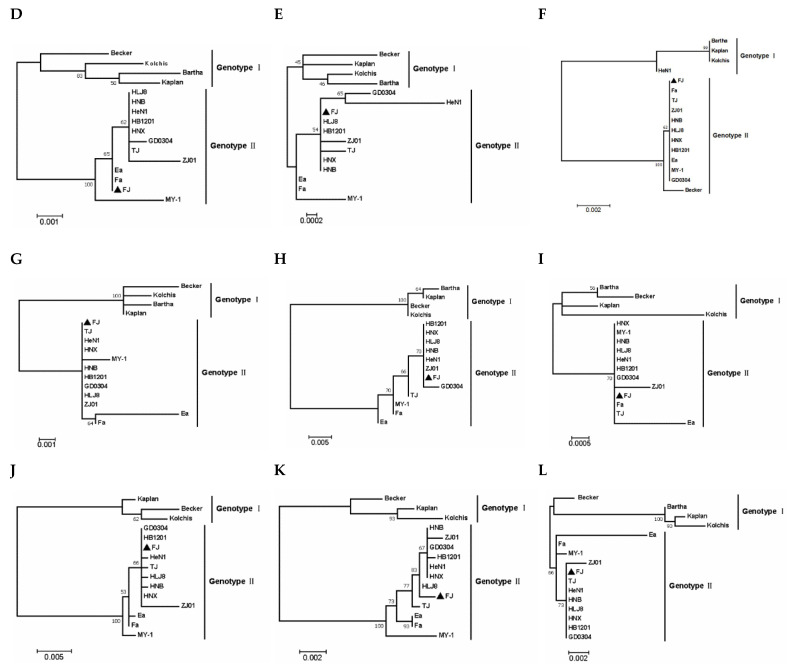
Phylogenetic analysis based on nucleotide sequences of PRV virulence-related and immunogenicity genes: (**A**) gB, (**B**) gC, (**C**) gD, (**D**) gG, (**E**) gH, (**F**) gL, (**G**) gM, (**H**) gN, (**I**) TK, (**J**) gI, (**K**) gE and (**L**) PK. The tree was constructed with the same method described in Figure 3.

We selected the coding sequences of 12 genes related to immunogenicity and virulence of PRV, including TK, PK, gB, gC, gD, gG, gH, gL, gM, gN, gI and gE genes and analyzed them using the ML method of the MEGA 7.0 software. Except for gL, the phylogenetic trees of all genes produced the typical genotype I and genotype II branches, while the gL evolutionary tree showed that the Chinese epidemic mutant HeN1 strain belonged to the European and the North American genotype I. The Becker strain belonged to genotype II. All the above genes of the FJ strain were located in the large genotype II branch, and its gB, gC, gD, gH, gL, gM, gN, TK, gI, and PK genes were in the same branch as other Chinese mutants. Its gG gene was assigned to the same branch with the classical Chinese PRV Fa and Ea strains’ gG gene, while its gE gene was assigned to a relatively independent branch. All the genes of the MY-1 strain were located in the large branch of genotype II, except for TK, gL, gM, and gN; the other genes were located in a single branch compared with Chinese strains. The selected PRV FJ genes were far away from the other country’s strains, and they were very close to the Chinese mutants; and, the above-mentioned immunogenicity and virulence-related genes were not significantly different from the previous PRV variants (Figure 4).

### 3.4. Recombination Analyses

**Figure 5:**
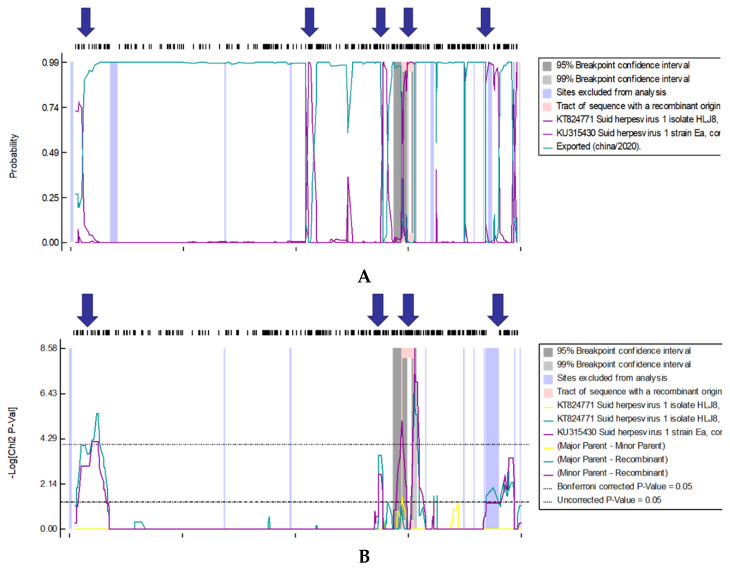
Putative recombination events in the PRV FJ strain complete genome. Recombination sites are marked by a blue arrow. The region with a pink background represents the potential recombination region. (**A**): BootScan method; (**B**): RDP method.

We compared the FJ strain with the 15 PRV reference genome sequences using the RDP4 software (http://web.cbio.uct.ac.za/~darren/rdp.html, accessed on 14 October 2021); we predicted the recombination possibilities of the strain using Bootscan, LARD, 3seq, PhylPro, Maxchi, SiScan, and Chimaera algorithms. We detected several recombination signals for the FJ genome sequence (Figure 5). The major backbone of the FJ strain was the HLJ8 strain; the minor backbone was the Ea strain. We analyzed the potential recombination events of the FJ complete genome sequence using the above-mentioned algorithms; the *p* value of each algorithm was <10^−3^. The predicted recombination sites were between 1694 and 1936 bp, between 101,113 and 102,660 bp, and between 107,964 and 11,148,1 bp; four algorithms supported the recombination events in each segment. Among them, two algorithms in the 1694–1936 bp section showed that the recombination event was credible; one algorithm in the 101,113–102,660 bp section showed that the recombination event was credible; and four algorithms in the 107,964–111,481 bp section showed that the recombination event was credible (Table 5).

## 4. Discussion

Due to the large number of genes in the PRV complete genome, the high content of GC bases, and the presence of more than 900 nucleotide repeat sequences, it is relatively difficult to sequence the complete genome; research in the PRV gene function and comparative genomics had been somewhat restricted. In 2011, American researchers were the first to obtain and to publish the complete genome sequences of some representative PRV strains such as Bartha, Kaplan, and Becker using Illumina second-generation sequencing technology [3]. With the popularity of second-generation high-throughput sequencing around the world, the complete genome sequences of PRV isolates from various regions have been published in China since 2014 [15]. The advantage of second-generation sequencing is that segmented sequencing can be used to ensure the accuracy of sequencing results; the cost of sequencing at this stage is very low; and the DNA samples do not need to be of very high quality to sequence. However, there are several disadvantages, including that the sequencing time is very long; the content of GC bases in PRV genomes is very high so that it is hard to completely sequence the genomes at one time; every sequencing of a complete genome will generate many gap sequences, which need to be amplified and filled by multiple pairs of primers; and the sequencing technology still needs to be innovated. In recent years, with the advent of the third-generation PacBio RSII gene sequencer, long and complex sequencing has become very convenient.

In this study, we combined second- and third-generation sequencing, an approach that provides the benefits of third-generation sequencing efficiency, ultra-long reading length, short sequencing cycle, no base preference, and no gap sequences. In addition, this approach allows the sequencing of complex structures at one time, and it makes use of MGISEQ-2000 s generation sequencing to make up for the shortcomings of low third-generation sequencing flux and manual correction of sequencing errors. After sequencing, we assembled the PRV FJ complete genome quickly, accurately, and completely. The PRV FJ complete genome was 143,703 bp, had a G&C bases content of 73.67%, and encoded 70 ORFs. The length and the structural range of the complete genome sequence are consistent with the previously tested Chinese epidemic mutant HNX strain (full length = 142,294 bp, G&C bases content = 73.56%, encoding 70 ORFs) [16]; the HNB strain (full length = 142,255 bp, G&C bases content = 73.61%, encoding 70 ORFs) [17]; the TJ strain (full length = 143,642 bp, encoding 67 ORFs) [15]; and the HeN1 strain (full length = 141,803 bp, G&C bases bases content = 73.3%, encoding 69 ORF) [18]. Thus, our FJ strain sequencing results are reliable.

Among the PRV genes we selected for phylogenetic tree analysis, gB, gD, gH, gL, and gK are necessary to ensure their replication, growth, and proliferation in cells [19]. As PRV immunogenicity-related proteins, gB, gC, and gD can induce neutralizing antibody production [20]. The proteins expressed by gH and gL, gE and gI, and gM and gN genes can form heterodimers, which are related to virus infection and immune escape [21]. TK, PK, gE and gI are virulence-related genes. Single or multiple deletions or insertion mutations in these genes affect the virulence of PRV [22,23]. The gG gene encodes the only protein component released by PRV outside the virus; it was released out of the cell by protease hydrolysis, and it was disconnected from the virion after passing through the cell membrane [24]. Among the above-mentioned genes, only the gG gene of the PRV FJ strain was located in the subbranch of the classic Chinese strains identified before 2011. The reason is that the 244th base of the gG gene of the FJ strain, classic Chinese strain and other country’s strains is ‘T’, while the corresponding base of the Chinese epidemic mutant is ‘C’. The other genes were in the same subbranch with the mutants in China. Therefore, the results showed that the FJ strain has also been a common variant in China in recent years, and the genetic variation has been stable up to now.

Through the detection of the complete genome recombination events of the PRV FJ strain, we found recombinant signals in the 1694–1936, 101,113–102,660, and 107,964–111,481 bp regions, indicating that recombination occurred in the corresponding regions of the HLJ8 and the Ea strains. According to the location of coding sequence annotations in Table 3, we found the recombinant region was located in the non-coding region of UL and IRs of the FJ strain, so there is no recombination mutation event in the coding sequence. Non-coding regions in virus genomes have a variety of functions. For example, a non-coding region of Japanese encephalitis virus antagonizes the interferon response by blocking interferon regulatory factor 3 transport [25]; the replication of Marburg virus can be regulated by its non-coding region [26]. Influenza virus infection can be regulated by its non-coding region [27]. The non-coding region of the Epstein–Barr virus (EBV) plays an important role in the life cycle and the pathogenesis of EBV [28]. Natural recombination between different PRV strains has been common, as authors have reported, although the mechanism is unclear [29].

However, gene recombination in the non-coding region may affect the ability of PRV to induce interferon-beta promoter activity and regulate viral messenger RNA (mRNA) [30,31]. The major backbone strain for the recombination event was HLJ8, which is an epidemic variant strain that was isolated in China after 2011, while the minor backbone strain was Ea, a classic strain that was isolated in China in the 20th century. Hence, the FJ strain might have the ability to recombine with the Chinese epidemic variant strain and the classical strain. With regard to the cause of the natural recombination phenomenon, we speculate that the FJ wild type strain may have arisen due to natural recombination in pigs when they were immunized with the commercial vaccine with the Ea strain as the parent strain. During large-scale importation of breeding pigs in this pig farm, the cross-provincial transportation of breeding pigs caused some pigs with latent PRV infection in other areas, so it was difficult to show positive results accurately during PRV detection, while these pigs are still traded in the market. After the infection of the FJ strain, gene recombination might occur among PRV in the host.

## Figures and Tables

**Table 1:** Complete genome sequence information of pseudorabies viruses in the National Center of Biotechnology Information databases.

No.	Strain Name	Accession Number	Country	Isolation Date
1	Becker	JF797219.1	USA	1970
2	Bartha	JF797217.1	Hungary	1961
3	Ea	KU315430.1	China	1990
4	Fa	KM189913.1	China	2012
5	GD0304	MH582511.1	China	2015
6	HB1201	KU057086.1	China	2012
7	HeN1	KP098534.1	China	2012
8	HLJ8	KT824771.1	China	2012
9	HNB	KM189914.3	China	2012
10	HNX	KM189912.1	China	2012
11	Kaplan	JF797218.1	Hungary	1959
12	Kolchis	KT983811.1	Greece	2010
13	MY-1	AP018925.1	Japan	2015
14	ZJ-01	KM061380.1	China	2012
15	TJ	KJ789182.1	China	2012

**Table 2:** Nucleotide sequence coordinates and lengths are given relative to the genome sequence of PRV FJ strain.

Region	Location	Length (bp)
UL	1–101,012	101,012
IRs	101,013–117,681	16,669
US	117,682–127,034	9353
TRs	127,035–143,703	16,669

**Table 3:** Annotation of each open reading frame of from the PRV FJ complete genome.

Protein Name	Location of ORF (bp)	Length (aa)	Function
UL56	754–1377	207	Possibly vesicular trafficking
ICP27	1932–3017	361	Gene regulation; early protein
gK	3096–4034	312	Viral glycoprotein K; type III membrane protein
UL52	3989–6895	968	DNA replication; primase subunit of ULS/UL8/UL52 complex
UL51	6882–7613	243	Tegument protein
dUTPase	7812–8621	269	dUTPase
gN	8542–8841	99	Glycoprotein N; type I membrane protein; complexed with gM
VP22	8879–9619	246	Interacts with C-terminal domains of gE and gM; tegument protein
VP16	9683–10,924	413	Gene regulation (transactivator); egress (secondary envelopment); tegument protein
VP13/14	11,034–13,250	738	Viral egress (secondary envelopment); tegument protein
VP11/12	13,269–15,356	695	Possibly gene regulation; tegument protein
gB	15,905–18,649	914	Viral entry (fusion); cell–cell spread; glycoprotein B; type I membrane protein
ICP18.5	18,520–20,688	722	DNA cleavage and encapsulations (terminase); associated with UL15, UL33 and UL6
ICP8	20,836–24,378	1180	DNA replication-recombination; binds single-stranded DNA
UL30	24,677–27,823	1048	DNA replication; DNA polymerase subunit of UL30/UL42 complex
UL31	27,744–28,559	271	Viral egress (nuclear egress); primary virion tegument protein; interacts with UL34
UL32	28,552–29,967	471	DNA packaging; efficient localization of capsids to replication compartments
UL33	29,966–30,322	118	DNA cleavage and encapsidation; associated with UL28 and UL 15
UL34	30,494–31,279	261	Viral egress (nuclear egress); primary virion envelop protein tail-anchored type II nuclear membrane protein; interacts with UL31
VP26	31,334–31,645	103	Capsid protein
VP1/2	32,057–41,644	3195	Large tegument protein; interacts with UL37 and UL19
UL37	41,682–44,441	919	Tegument protein; interacts with UL36
VP19c	44,498–45,604	368	Capsid protein; forms triplexes together with ULl8
RR1	45,941–48,307	788	Nucleotide synthesis; large subunit of ribonucleotide reductase
RR2	48,317–49,228	303	Nucleotide synthesis; small subunit of ribonucleotide reductase
vhs	49,843–50,940	365	Gene regulation (inhibitor of gene expression); virion host cell shutoff
UL42	51,069–52,226	385	DNA replication; polymerase accessory subunit of UL30/UL42 complex
UL43	52,286–53,407	373	Unknown; type III membrane protein
gC	53,474–54,937	487	Viral entry (virion attachment); glycoprotein C; type I membrane protein; binds to heparan sulfate
UL26.5	55,233–56,093	286	Scaffold protein; substrate for UL26; required for capsid formation and maturation
VP24	55,233–56,831	532	Scaffold protein; proteinase; required for capsid formation and maturation
UL25	56,883–58,493	536	Capsid-associated protein; required for capsid assembly
UL24	58,592–59,107	171	Unknown; type III membrane protein
TK	59,100–60,062	320	Nucleotide synthesis; thymidine kinase
gH	60,198–62,255	685	Viral entry (fusion); cell–cell spread; glycoprotein H; type I membrane protein; complexed with gL
UL21	64,012–65,613	533	Capsid-associated protein
UL20	65,720–66,217	165	Viral egress; type III membrane protein
VP5	66,306–70,298	1330	Major capsid protein; forms hexons and pentons
VP23	70,473–71,363	296	Capsid protein; forms triplexes together with UL38
UL17	72,739–74,538	599	DNA cleavage and encapsidation
UL16	74,565–75,551	328	Possibly virion morphogenesis
UL15	71,546–72,687 75,572–76,691	753	DNA cleavage and encapsidation; terminase subunit; interacts with UL33 UL28, and UL6
UL14	76,690–77,169	159	Virion morphogenesis
VP18.8	77,139–78,314	391	Protein-serine/threonine kinase
AN	78,280–79,731	483	DNA recombination; alkaline exonuclease
UL11	79,689–79,880	63	Viral egress (secondary envelopment); membrane-associated tegument protein
gM	80,309–81,490	393	Viral egress (secondary envelopment); glycoprotein M; type III membrane protein; C terminus interacts with UL49; inhibits membrane fusion in transient assays; complexed with gN
OBP	81,489–84,023	844	Sequence-specific ori-binding protein
UL8	84,020–86,086	688	DNA replication; part of ULS/UL8/UL52 helicase-primase complex
UL7	86,288–87,088	266	Virion morphogenesis
UL6	86,979–88,916	645	DNA packaging Capsid protein; portal protein; docking site for terminase
UL5	88,915–91,470	851	DNA replication; part of ULS/LJL8/UL52 helicase-primase complex; helicase motif
UL4	91,528–91,965	145	Nuclear protein
UL3.5	92,141–92,809	222	Possibly virion morphogenesis
UL3	92,806–93,540	244	Nuclear protein
UNG	93,597–94,568	323	Uracil-DNA glycosylase
gL	94,546–95,016	156	Viral entry; cell–cell spread; glycoprotein L; membrane-anchored via complex with gH
ICP0	96,248–97,348	366	Gene regulation (transactivator of viral and cellular genes); early protein
ICP4	103,130–107,544	1471	Gene regulation; immediate early protein
ICP22	116,146–117,339	397	Gene regulation
PK	118,467–119,471	334	Minor form of protein kinase (53-kDa mobility); viral egress (nuclear egress); major form of protein kinase (41-kDa mobility)
gG	119,531–121,030	499	Cell–cell spread; secreted; glycoprotein G
gD	121,214–122,422	402	Viral entry (cellular receptor binding protein); glycoprotein D
gI	122,446–123,543	465	Cell–cell spread; glycoprotein I; type I membrane protein; complexed with gE
gE	123,647–125,386	579	Cell–cell spread; glycoprotein E; type I membrane protein; complexed with gI; C terminus interacts with UL49
US9(11K)	125,444–125,740	98	Protein sorting in axons; type II tail-anchored membrane protein
US2(28K)	125,994–126,764	256	Possibly envelope associated

Abbreviations: aa, amino acids; ORF, open reading frame.

**Table 4:** Complete gene sequence nucleotide homology analysis.

Virus Strain	Nucleotides Homology (%)															
	MY-1	FJ *	Bartha	Kaplan	Becker	TJ	ZJ01	HNX	Fa	HNB	HeN1	HLJ8	Kolchis	HB1201	Ea	GD0304
MY-1																
FJ *	99.0															
Bartha	95.4	95.7														
Kaplan	95.6	96.0	99.4													
Becker	95.4	95.7	98.6	98.9												
TJ	99.0	100	95.7	96.0	95.7											
ZJ01	99.0	99.9	95.6	95.9	95.6	99.9										
HNX	99.0	100	95.7	96.0	95.7	100	99.9									
Fa	98.9	99.7	95.7	96.0	95.6	99.7	99.7	99.7								
HNB	99.0	100.0	95.7	96.0	95.7	100.0	99.9	100.0	99.7							
HeN1	99.0	100.0	95.7	96.0	95.7	100.0	99.9	100.0	99.7	100.0						
HLJ8	99.0	100.0	95.7	96.0	95.7	100.0	99.9	100.0	99.7	100.0	100.0					
Kolchis	95.4	95.7	99.1	99.5	99.0	95.7	95.6	95.7	95.7	95.7	95.7	95.7				
HB1201	99.0	100.0	95.7	96.0	95.7	100.0	99.9	100.0	99.7	100.0	100.0	100.0	95.7			
Ea	98.9	99.7	95.7	96.0	95.6	99.7	99.7	99.7	100.0	99.7	99.7	99.7	95.7	99.7		
GD0304	99.0	100.0	95.7	96.0	95.7	100.0	99.9	100.0	99.7	100.0	100.0	100.0	95.7	100.0	99.7	

Note. “*” indicates that this PRV strain is the target PRV strain.

**Table 5:** Analysis of PRV FJ recombination events with different algorithms; the numbers represent the *p* value of each algorithm.

Position (bp)	Method and *p* Value						
	Bootscan	Maxchi	Chimaera	SiScan	PhylPro	LARD	3Seq
1694–1936	2.31 × 10^−11^ *	3.50 × 10^−9^ *	NS	2.27 × 10^−2^	1.05 × 10^−2^	NS	NS
101,113–102,660	6.54 × 10^−5^ *	6.26 × 10^−3^	NS	5.67 × 10^−3^	1.82 × 10^−3^	NS	NS
107,964–111,481	9.87 × 10^−10^ *	9.81 × 10^−9^ *	NS	1.93 × 10^−5^ *	1.68 × 10^−4^ *	NS	NS

Note. NS, does not support reorganization events; * *p* < 10^−3^^.^

## Data Availability

The complete genome of the virus described in detail here was deposited in GenBank under the following Accession Numbers: MW286330.
